# Supplementary material for: Spin relaxation of electron and hole polarons in ambipolar conjugated polymers
Source: Nat Commun. 2024 Jan 4;15:288. doi: 10.1038/s41467-023-43505-7 (PMC10767019; doi:10.1038/s41467-023-43505-7)
Supplement: Supplementary file 1 — Supplementary Information [file 41467_2023_43505_MOESM1_ESM.pdf]

Supplementary Information: Spin Relaxation of Electron and Hole Polarons in Ambipolar Conjugated Polymers

October 25, 2023

Author list

Remington L. Carey[†,1], Samuele Giannini[†,2,5], Sam Schott[1], Vincent Lemaur[2], Mingfei Xiao[1], Suryoday Prodhan[3], Linjun Wang[4], Michelangelo Bovoloni[2], Claudio Quarti[2], David Beljonne[2], Henning Sirringhaus[*,1]

[1] University of Cambridge, Cavendish Laboratory, Cambridge, CB3 0HE, United Kingdom

[2] University of Mons, Laboratory for Chemistry of Novel Materials, 7000 Mons, Belgium

[3] University of Liverpool, Department of Chemistry, Liverpool L69 3BX, United Kingdom

[4]Key Laboratory of Excited-State Materials of Zhejiang Province, Department of Chemistry, Zhejiang University, Hangzhou 310058, China

[5]Present address: Institute of Chemistry of OrganoMetallic Compounds, National Research Council (ICCOM-CNR), I-56124 Pisa, Italy

[†] Equal contribution

[*] Correspondence: hs220@cam.ac.uk

Supplementary Note 1

Measurement of background signals in FI-ESR

In ESR studies, the test tubes used for measurement have extremely low amounts of magnetic impurities to prevent spurious contributions to the ESR signal. However, even small amounts of impurities can be detected at low temperatures due to the Curie susceptibility of such materials. We always use the highest-purity tubes available for our ESR studies, but occasionally even those have detectable levels of impurities at low temperatures. In such cases, the spurious signal can be removed by measuring our FI-ESR devices at non-zero gate voltage (i.e., under the desired measurement conditions) and subtracting from that signal a signal obtained by measuring at Vg = 0 V (which is the background signal). We followed this procedure for the data presented here. We note that care must be taken in doing so because the microwave frequency may change between measurements, meaning the resonance condition equation must be used to properly align the two spectra before subtraction.

Supplementary Note 2

Extraction of Spin Lifetimes

As mentioned in the main text, spin lifetimes were extracted using the same method as in our previous work.[1]. We briefly summarize that method here.

In most ESR experiments on organics, the sample of interest may be treated as a collection of spins all described by the same Hamiltonian. This Hamiltonian consists primarily of the spin-Zeeman interaction describing the coupling between a spin S and the externally applied magnetic field B. The sample's interaction with the field manifests as the paramagnetic bulk becoming magnetized at equilibrium, a process which occurs over a relatively quick timescale. A continuous interaction is achieved by applying a oscillatory microwave field with amplitude B_{mw} and frequency ω_{mw} to the sample, which acts to repeatedly push the sample out of equilibrium each time it aligns. This repeated absorption of microwave energy is detected as the ESR signal. The characteristic timescale the sample takes to become unaligned with the quantization axis (set by the external field) is the longitudinal relaxation time, T_1, while the characteristic timescale over which coherent spins no longer precess around the quantization axis with each other is the transverse relaxation time, T_2. The power absorbed over one cycle of the microwave field can be written as follows:

$$\overline{L}(B) = \frac{B_{\mathrm{mw}}^2 \chi_0 \gamma_{\mathrm{e}} B \omega_{\mathrm{mw}}}{\sqrt{1+\gamma_{\mathrm{e}}^2 B_{\mathrm{mw}}^2 T_1 T_2}} \underbrace{\frac{1}{\pi\rho} \frac{\rho^2}{\rho^2 + (\gamma_{\mathrm{e}} B - \omega_{\mathrm{mw}})^2}}_{\mathscr{L}}, \tag{1}$$

where χ_0 is the static magnetic susceptibility of the sample and

$$\rho \equiv \frac{\sqrt{1+\gamma_{\mathrm{e}}^2 B_{\mathrm{mw}}^2 T_1 T_2}}{\gamma_{\mathrm{e}} T_2} \tag{2}$$

is the half-width-at-half-height of the normalized Lorentzian function $\mathscr{L}$. It is clear that absorption will be greatest when the resonance condition $\omega_{\mathrm{mw}} = \omega_{\mathrm{L}}$ is fulfilled.

The previous expression describes the resonance signal if all spins can be treated equivalently. This may not the case, however, and a more general function is required. It is often assumed that a Gaussian spread of resonance positions can be used to describe a collection of spins in such a case:

$$\mathcal{G}(B) = \frac{1}{\sqrt{2\pi B_\nu^2}} \exp\left[-\frac{(B - \omega_{\mathrm{mw}}/\gamma_{\mathrm{e}})^2}{2B_\nu^2} \right]. \tag{3}$$

The overall lineshape then follows as a convolution of this Gaussian spread with the Lorentzian resonance signal,

$$\mathcal{V} = \int_{-\infty}^{\infty} \mathcal{G}(B') \mathcal{L}(B - B') dB', \tag{4}$$

which is known as a *Voigtian*. This is normally taken as a more general function to fit, and the Gaussian spread is negligible in cases where all spins are indeed equivalent.

There are two experimental factors that must also be considered before fitting. The first is that the detection diode is proportional to the square root of the microwave power, which modifies the absorption

equation so that it is only linear in the amplitude of the microwave field. The second is that a lock-in amplifier is used to increase sensitivity of the signal. The effect of a lock-in amplifier is to detect the derivative absorption signal with respect to the Zeeman field; this is mathematically equivalent to adding a sinusoidal modulation signal to the field. Care must be taken in sitting the amplitude of the modulation signal, as signals that are too large can create artifacts in the signal. To correct for this, a routine in MATLAB was used for the data described here. By exploiting the fact that the diode's response to the signal can be written as a Fourier transform of the signal, and that phase-sensitive detection has no variance with time, the routine models the recorded spectrum as a convolution between a modulation kernel and the absorption signal. The resultant signal we fit has the form

$$\mathcal{S}(B) = \int_{-\infty}^{\infty} K_1(B)\mathcal{V}(B - B')dB', \tag{5}$$

where

$$K_n(B) = i^2 \int_{-\infty}^{\infty} J_n\left(\frac{B_{\text{mod}}B'}{2}\right)e^{iBB'}dB' \tag{6}$$

is defined by the nth-order Bessel function. This is the equation to which we fit the data in order to obtain the relaxation times T_1 and T_2, as well as resonance positions and root-mean-square of the convolving Gaussian spread (if any).

Supplementary Note 3

Low-temperature spectra

In Fig. 1, we show ESR data for all three systems at 10 K and 100 K for comparison to the data presented in Figure 2 in the main text. It is important to note that differences in signal-to-noise ratios between materials may represent true material differences and/or differences in measurement conditions. The intensities of the resonance signals of both holes and electrons in DPPT-TT (Fig. 1(a)) at 10 K are particularly low, thus resulting in the comparatively large, visible signal from background impurities mentioned in the previous supplementary note. Fitting these spectra was not an issue, as evidenced by the reasonable error bars shown for T_1 and T_2 at 10 K in Figure 3(a) in the main text.

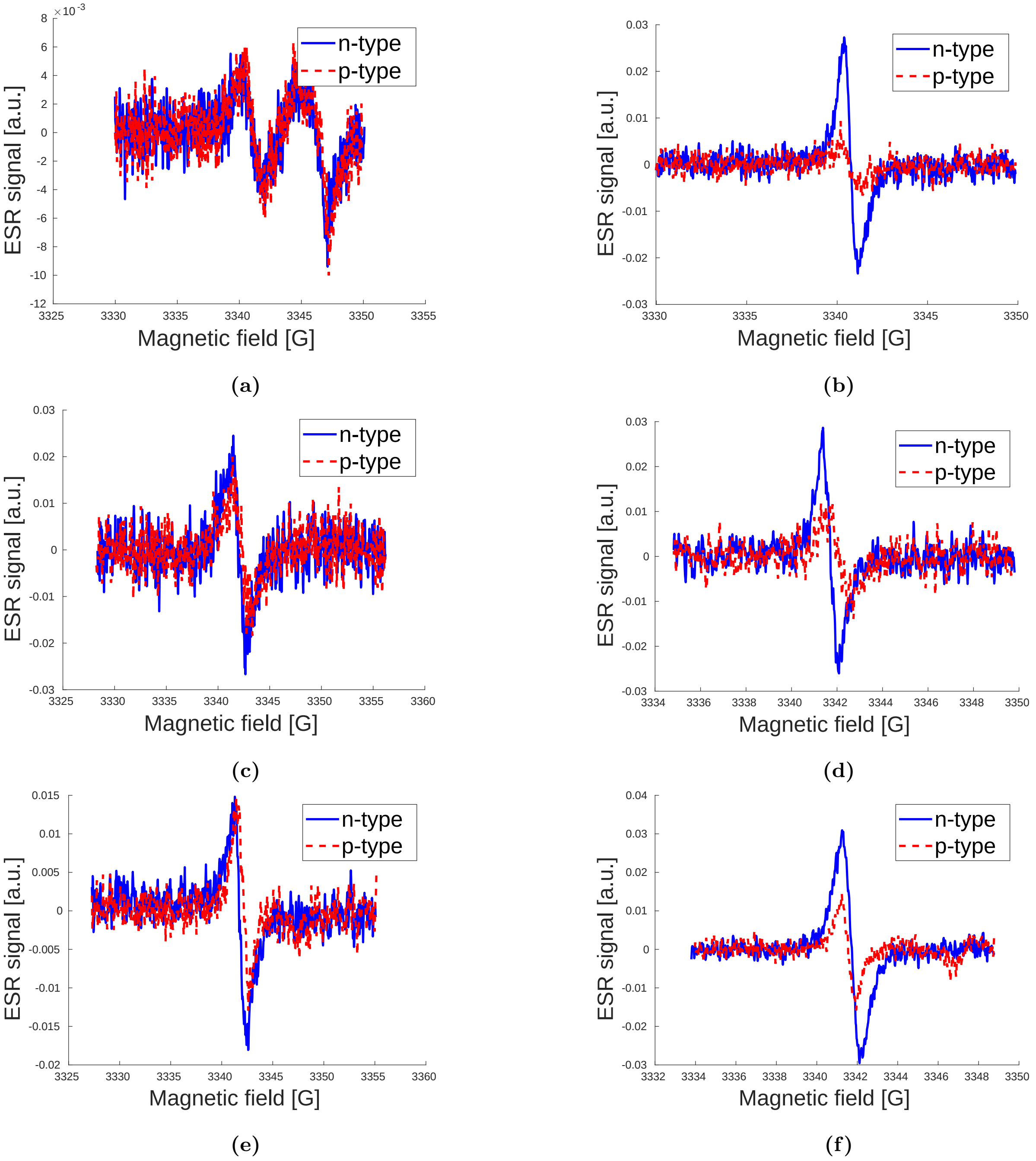

Supplementary Figure 1: Low- and mid-temperature data for all three systems. (a),(b): DPPT-TT at 10 and 100 K. **(c),(d)**: AN data at 10 and 100 K. **(e),(f)**: NN data at 10 and 100 K. Variance in signal-to-noise ratios is due to different integration times being used.

Supplementary Note 4

Longitudinal and transverse relaxation times

The full temperature dependencies of T_1 and T_2 for electron and hole polarons in all three systems in shown in Fig. 2. As reported in the main text, all three systems show the inhomogeneous broadening, motional narrowing, and spin-shuttling regimes. Because T_1 monotonically decreases with temperature, only T_2 is shown in the main text.

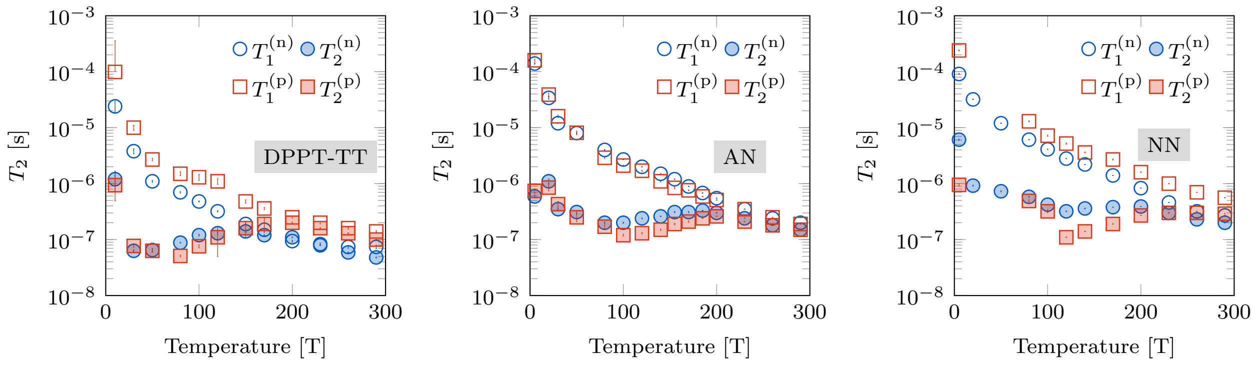

(a) **(b)** **(c)**

Supplementary Figure 2: Full temperature dependence of n- and p-type polaronic relaxation times. All three polymers studied show the three regimes of spin relaxation: inhomogeneous broadening, motional narrowing, and spin-shuttling.

Supplementary Note 5

DFT calculations on molecular systems

The DPPT-TT polymer (Supplementary Fig. 3) is the main subject of our computational analysis. This copolymer is composed of alternating electron-deficient (diketopyrrolopyrrole derivative - DPPT) and electron-rich (thiophene-thieno[3,2-b]thiophene - TT) moieties along the backbone.

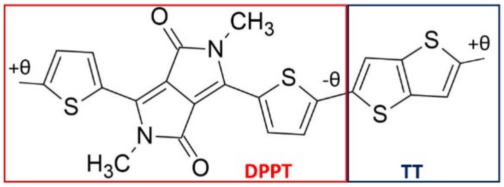

Supplementary Figure 3: The DPPT-TT polymer repeat unit used for our computational analyses. The red and blue rectangles highlight the DPPT (donor) and TT (acceptor) fragments, respectively.

In Supplementary Fig. 4, we report the charge distribution and the torsional angle of a DPPT-TT pentamer. The atomic charges were obtained by fitting the electrostatic potential (ESP charges) calculated at the LC-ωhPBE/6-31G(d,p) level on the neutral, positively, and negatively charged DPPT-TT pentamer after optimization of the corresponding charge configuration. For our pentamer, we found that the charge density of the electron wavefunction is different than that of holes: The radical cation is delocalized only over a single DPP unit and its two connected thiophene units, while the radical anion is delocalized over a central DPP unit, its two adjacent TT units, and still has some amplitude on the adjacent DPP units. The more pronounced delocalization of the electron is also reflected in a slightly more extended planarization of the bond angles along the chain, as shown in Supplementary Fig. 4(b). The electron polaron planarizes four T-TT bonds, while the hole polaron only planarizes two such bonds.

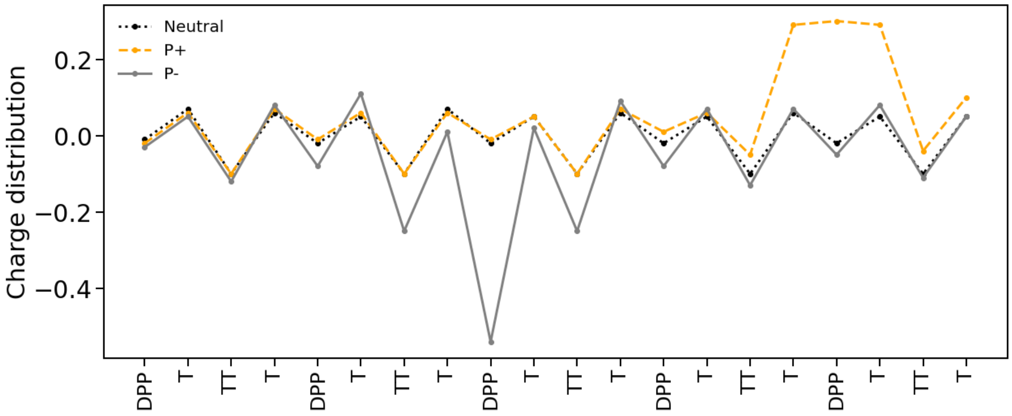

(a)

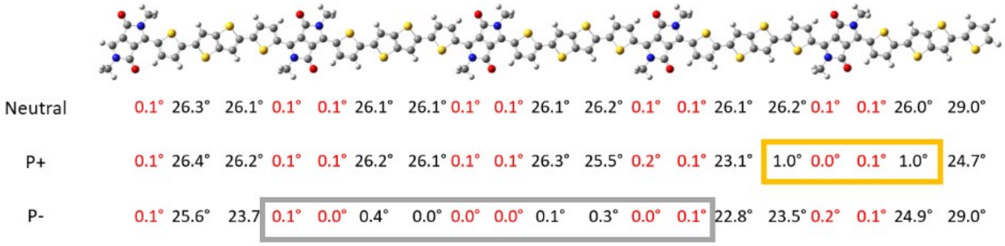

(b)

Supplementary Figure 4: Charge distribution. **(a)**: Localization of the neutral (dotted black line), radical cation P+ (dashed orange line), and radical anion P- (grey line) wavefunctions simulated for a chain of five repeat units. **(b)**: Torsion angle of neutral, positive, and negative pentamers.

Supplementary Note 6

Model Hamiltonian and coarse graining

To study transport properties and to calculate intra-chain electron and hole mobilities, the DPPT-TT copolymer was coarse-grained as a one-dimensional array of N DPPT donor (D) and TT acceptor units (A), i.e., DADADA (see Fig. 3). Each monomer of (D or A unit) k was associated with a single frontier molecular orbital $|k\rangle$. The HOMO orbitals of DPPT and TT units were then used to investigate hole transport, while the LUMO orbitals were used for electron transport. The suitability of such an approximation is verified using explicit ab-initio periodic DFT calculations in the next section. DFT calculations performed on the neutral ground-state structure of a DPPT-TT pentamer revealed that the bond angle associated with the DPP-T linkage is planar ($\theta_{\text{DPP-T}} = 0.1°$), while the T-TT linkages exhibit a torsion angle of 26° (see Supplementary Fig. 4). This further supports the copolymer partition represented in Supplementary Fig. 3.

We coarse-grained the polymer chain using a Su–Schrieffer–Heeger (SSH)-type model Hamiltonian, which considers both local and non-local electron–phonon (e-ph) couplings.[2] Similar to our previous works,[3, 4] the electronic structure of the polymer can be described by the following total (nuclear + electronic) Hamiltonian,

$$\mathscr{H} = \mathscr{H}_e + \mathscr{H}_n, \tag{7}$$

with the electronic part written as

$$\mathscr{H}_e = \sum_{k=1}^{N} (\epsilon_k + \alpha_{k,1} x_{k,1} + \alpha_{k,2} x_{k,2}) |k\rangle \langle k| + \sum_{k=2}^{N} J|\cos\theta_{k,k-1}|[|k\rangle \langle k-1| + |k-1\rangle \langle k|], \tag{8}$$

and the nuclear part as

$$\mathscr{H}_n = \sum_{k=1}^{N} \frac{1}{2}\left[m_{k,1} v_{k,1}^2 + K_{k,1} x_{k,1}^2 + m_{k,2} v_{k,2}^2 + K_{k,2} x_{k,2}^2 + I_k \omega_{k,3}^2\right] + \sum_{k=2}^{N} \frac{K_\theta}{2} (\theta_{k,k-1} - \theta_{\text{eq.}})^2. \tag{9}$$

Here, ϵ_k is the on-site energy of the donor/acceptor moiety in the neutral ground-state equilibrium geometry and J is the electronic (hole or electron) transfer coupling modulated by a cosine function with angle $\theta_{k,k-1}$ describing the torsion between the DPPT and TT units (see Fig. 3). Maximum coupling is achieved when $\theta_{k,k-1} = 0$ or 180°.

The dynamics of the nuclei are described by three effective, classical, harmonic vibrational degrees of freedom: $x_{k,1}$, $x_{k,2}$, and $\theta_{k,k'}$. The first two represent a high- and low-frequency intra-monomer mode, respectively, and account for the change in monomer geometry upon addition of excess holes or electrons. The k-th monomer energy is linearly modulated with these parameters by a local electron-phonon coupling constant ($\alpha_{k,1}$ and $\alpha_{k,2}$). This, in turn, is related to the relaxation energy $\lambda_{k,1(2)}^{\text{rel.}}$ (also called the binding energy of the polaron[5]) by $\alpha_{k,1(2)} = \sqrt{2K_{k,1(2)}\lambda_{k,1(2)}^{\text{rel.}}}$. The force constant of the harmonic oscillator is $K_{k,1(2)} = m_{k,1(2)}\omega_{k,1(2)}^2$, where $m_{k,1(2)}$ is the reduced mass of the monomer k and $v_{k,1(2)}(\omega_{k,1(2)})$ corresponds to the linear velocity (angular frequency).

The third mode, $\theta_{k,k'}$, represents the torsion between successive monomer units k and k' along the polymer axis and modulates the nearest-neighbor electronic coupling J so that the non-local electron-phonon coupling is accounted for. $\theta_{\text{eq.}}$ is the equilibrium value of the inter-monomer torsion angle in the charge-neutral polymer chain. I_k is the moment of inertia of monomer k, $\omega_{k,3}$ is the angular velocity of the third mode, and K_θ is the inter-monomer torsional stiffness constant.

We remark that the Hamiltonian in eq. 7 is similar to the one used before to study intra-chain transport in P3HT polymers[4] and IDTBT copolymers.[3] The main difference here is related to the steps taken to parameterize elements of the Hamiltonian (which will be described in the following sections) and the presence of two modes modulating the local e-ph couplings.

Supplementary Note 7

Electronic band structure of 1D periodic systems

The Hamiltonian in eq. 7 gives a rough 1D representation of a periodic copolymer with DPPT and TT sites present in the same unit cell, both contributing their HOMOs (LUMOs) to hole (electron) transport. The following calculations demonstrate that this minimal representation successfully describes the two highest occupied valence bands (VB and VB-1) and the two lowest unoccupied conduction bands (CB and CB+1) of the real DPPT-TT copolymer and provide numerical values for the parameters ϵ_k and J. We start from ab-initio, periodic DFT simulations of planar DPPT-TT ($\theta = 0°$) using the LC-ωhPBE functional and 6-31G(d,p) basis set. Calculations are performed with the Gaussian 16 program employing periodic boundary conditions. The valence and conduction bands are reported with orange and blue lines in Supplementary Fig. 5(a). To explain the VB (CB) dispersion obtained by DFT, we use a linear combination ($\tilde{\Psi}_{\text{tot.}} = C_{\text{A}}\tilde{\Psi}_{\text{A}} + C_{\text{D}}\tilde{\Psi}_{\text{D}}$) of Bloch states of the HOMOs (LUMOs) of donor and acceptor units (where a is the lattice vector):

$$\tilde{\Psi}_{\text{D}} = \frac{1}{\sqrt{N}} \sum_{R}^{N} e^{i\bar{k}R} \Psi_{\text{D}}(r - R) \tag{10}$$

$$\tilde{\Psi}_{\text{A}} = \frac{1}{\sqrt{N}} \sum_{R'} N e^{i\bar{k}(R' - a/2)} \Psi_{\text{D}}(r - R' - \frac{a}{2}). \tag{11}$$

In this representation, the VB (CB) (tight-binding) Hamiltonian becomes

$$\mathscr{H}(k) = \begin{bmatrix} \epsilon_{\text{D}} & 2iJ \sin \bar{k}\frac{a}{2} \\ -2iJ \sin \bar{k}\frac{a}{2} & \epsilon_{\text{A}} \end{bmatrix} \tag{12}$$

in which we can recognize the energy ϵ_k of the HOMOs (LUMOs) of D and A units and the related hole (electron) transfer couplings J. Solving the characteristic polynomial associated with the $\bar{k}$-dependent Hamiltonian gives access to the dispersion of VB (CB) and VB-1 (CB+1), represented with dashed red (blue) lines in Fig. 5(a). From this analysis we extracted site energies and couplings to insert into the model Hamiltonian (eq. 7); these values are reported in Supplementary Table 1.

To test the quality of the sinusoidal function modulating the electronic coupling J in eq. 7, we repeated periodic DFT calculations for structures rotated by 15, 30, 45, 60, and 90° around the dihedral angle between the DPPT and TT units. We then reconstructed the band structure in these different cases and computed the electronic coupling J (see Fig. 5(b)).

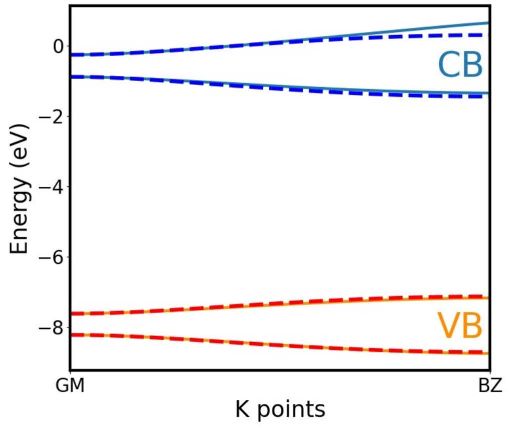

(a)

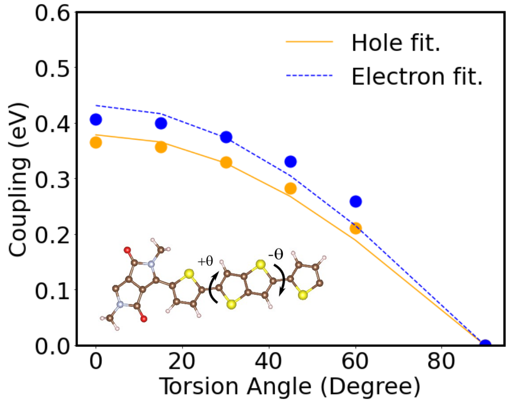

(b)

Supplementary Figure 5: Band structure properties. **(a)**: Band dispersion for $\theta = 0°$. Orange and cyan solid line represent the DFT-calculated dispersion of the valence (VB-1, VB) and conduction bands (CB, CB+1), respectively. Dispersions represented by the dashed lines were derived from solutions to the tight-binding problem (eq. 12) for the valence and conduction bands. **(b)**: Nearest-neighbor coupling J between DPPT and TT units as a function of torsion angle θ. Filled blue and orange circles represent values for electrons and holes, respectively, while the corresponding lines represent fits of these values to cosine functions.

Supplementary Table 1: Site energies and electronic couplings for DPPT and TT units. All values in eV.

Hole			Electron		
VB-1	VB		CB	CB+1	
ϵ_{TT}	ϵ_{DPPT}	$\lvert J^{\mathrm{hole}}\rvert$	ϵ_{DPPT}	ϵ_{TT}	$\lvert J^{\mathrm{elec}}\rvert$
-8.221[a]	-7.617[a]	0.365	-0.891	-0.264	0.407

[a] When performing hole transport simulations the sign of the site energies of DPPT and TT are inverted such that the excess hole relaxes preferentially on the DPPT unit. This is conveniently done to ensure that, along dynamics, the energy levels of the excess electron and hole are approximately populated according to $\exp(-E_i/k_{\mathrm{B}}T)$ where E_i are the excess electron or hole energy levels (see also the discussions in Refs. [6] and [7] on this point).

Supplementary Note 8

Relaxation energies and local electron-phonon couplings

The remaining parameters characterizing the coarsely grained Hamiltonian in eq. 7 are local electron-phonon couplings and related force constants. These values are connected to the polaron relaxation energies induced by the excess hole (electron) present on the polymer. To calculate these terms, we performed DFT optimization and extracted normal modes from the neutral, cationic, and anionic configurations of both DPPT and TT monomers in the gas phase (again using LC-ωhPBE/6-31g(d,p)). We used a displaced harmonic oscillator model in which we projected each intra-molecular normal mode of vibration on the vector describing the geometric changes between the neutral and charged (i.e., anionic, or cationic) state to partition the relaxation energies into mode contributions. The normal mode contributions to relaxation energies were computed with the MOMAP package[8] following a previously reported methodology.[9]

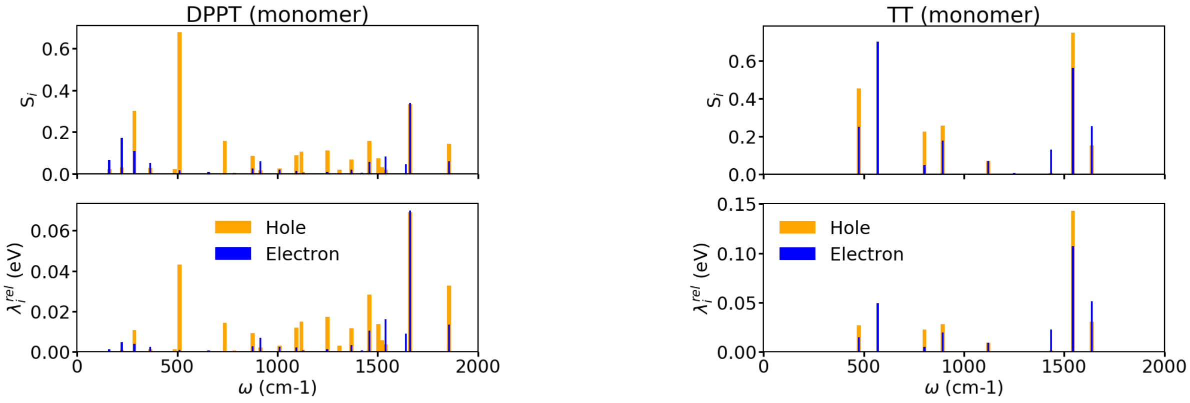

(a) **(b)**

Supplementary Figure 6: Relaxation energies. **(Top)**: Huang-Rhys factors (S) and **(bottom)** relaxation energies when going from the neutral to oxidized geometries (orange bars) and from the neutral to reduced geometries (blue bars) for **(a)** DPPT and **(b)** TT monomers, respectively.

In Supplementary Fig. 6, we can see that for both DPPT and TT units, hole and electron relaxation energies involve vibrational mode frequencies above k_BT. Thus, as done by other authors,[10] we assume that the full internal relaxation energy is carried by a single high frequency mode, $x_{k,1}$, in both DPP and TT units. This is characterized by an effective frequency $\hbar\omega^{\text{eff.}}$, which can be evaluated by weighing each frequency across the full spectrum in Supplementary Fig. 6 by the corresponding Huang-Rhys factor S_i, namely $\hbar\omega^{\text{eff.}} = \sum_i \hbar\omega_i S_i / \sum_i S_i$. We note in passing that S is related to the local e-ph coupling constant by $S = \alpha^2/2\hbar\omega K$ (where the subscript is dropped for simplicity). The results are reported in Supplementary Table 2.

On the other hand, the low frequency mode, $x_{k,2}$, is associated to an 'external' mode with an additional contribution from the environment to the reorganization energy (similar in spirit to the outer-sphere reor-

Supplementary Table 2: Relaxation energies and local e-ph coupling constants

	DPPT		TT	
	Electron	Hole	Electron	Hole
λ_k^{rel} [eV]	0.155	0.302	0.28	0.263
$\hbar\omega^{\text{eff.}}$ [cm^{-1}]	1032.5	1110.8	1062.8	956.5
$K_{k,1}$ [amu/ps^2] [a]	226972.8	262685.8	240447.5	194780.7
$\alpha_{k,1}$ [cm^{-1}/Å]	21780.8	32707.2	30130.8	26282.8

[a] According to what was done in Ref. [10], to calculate the force constant we assumed that $m_{(k,1)} = 6$ amu, which is the reduced mass of the C−C stretching coordinates that give the largest contribution to these modes (as shown in Fig. 5).

ganization energy in Marcus theory[5]). This external contribution was assumed to be either 100 and 150 meV, which is the typical range for reorganization energies found in many organic semiconductors.[11, 12] The frequency associated with this low-frequency mode was taken to be 40 cm^{-1} and was defined as the reduced mass of DPPT and TT units (326 and 140 amu, respectively). We verified that the results are not very sensitive to the frequency of the low frequency mode when varied from 40 to 20 cm^{-1}.

Supplementary Note 9

Ground-state torsion potential

The potential associated with the dihedral angle between DPPT and TT units in eq. 7 was computed using DFT at the same level of theory employed above and performing a relaxed scan at various θ on the DPPT-TT monomer at the LC-ωHPBE/6-31G(d,p) level. The result is reported with a black line in Supplementary Fig. 7. We fit each potential well within a simple harmonic oscillator approximation and this led to torsional stiffness constants of $K_\theta = 1753.9$ cm^{-1}/rad^2 and 1551.4 cm^{-1}/rad^2 around $\theta_{\text{eq.}} = 33$ and 157°, respectively. Using these values for the force constants and taking the masses as the reduced masses of the DPPT and TT units one can calculate the frequencies corresponding to the torsional degrees of freedom of both monoeric units. These are 25.5 and 36.5 cm^{-1}, respectively We also found asymmetry in the potential energy surfaces between syn and anti conformations, where the syn conformation was slightly higher in energy ($\Delta E \sim 0.025$ eV). We accounted for this asymmetry by considering a Boltzmann distribution of the torsional conformations in the neutral polymer chain (as explained in our previous work[4]), and we also kept track of the time-evolved inter-monomer torsion angles during the simulation.

The moment of inertia was evaluated as $I_k = m_k R^2$, where the masses were taken to be reduced masses of the DPPT and TT units. The distance R of the centers of mass of monomer units from the axes of rotation was evaluated by measuring the distance between the centers of mass of two randomly selected DPPT or TT units and the best fit line through all the conjugated atoms along a 2-nanosecond-long molecular dynamics evolution (NPT, p = 1 atm, room temperature, snapshots saved every 5 ps) and found to be 0.23 and 0.24 Å for DPPT and TT, respectively.

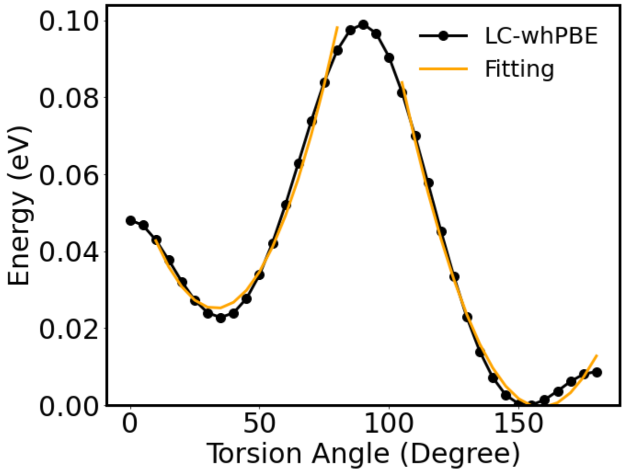

Supplementary Figure 7: DFT-calculated torsion potential between DPPT and TT units and related fitting. Details given in the text.

Supplementary Note 10

Potential influence of g factor anisotropy

The extracted B_{rms} values represent the average hyperfine coupling constant weighted by the square root of the number of molecules over which the wavefunction is spread. The hyperfine tensor can be formally expanded in orders of v/c, where v is the speed of the polaron and c is the speed of light. Doing so yields first-order contributions from the contact and dipolar terms (the usual hyperfine terms), and second-order contributions from spin-orbit interactions. Anisotropy in both couplings are therefore captured by our reported values, but of course we only report the average of these couplings. Because spin-orbit coupling is a second-order interaction, anisotropy in the g-factor will have a comparatively weak contribution to the extracted values; the dominant contributions will be those of the hyperfine interaction(s).

Supplementary Note 11

Wavefunction spread at different temperatures

We computed the spread of the charge-carrier wavefunction in time by representing the average population over several trajectories for all sites in Supplementary Fig 8. We can see how increasing the temperature increases the spreading of the wavefunction of holes and electrons alike, consistent with the fact that both charge types have higher mobility at higher temperatures. It is also interesting to note that the hole wavefunction at low temperatures remains essentially confined to a few sites around the central one (onto which the charge was initially relaxed). That is, small thermal fluctuations do not allow the hole to move very much; this is in contract to the electron wavefunction, which is more susceptible to nuclear motion.

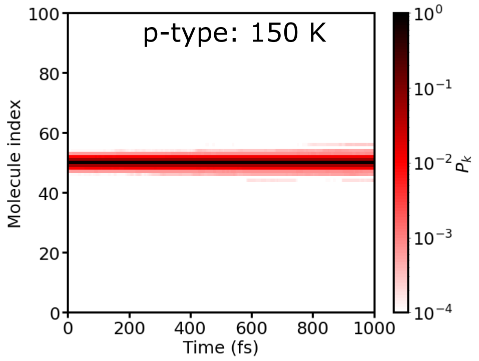

(a)

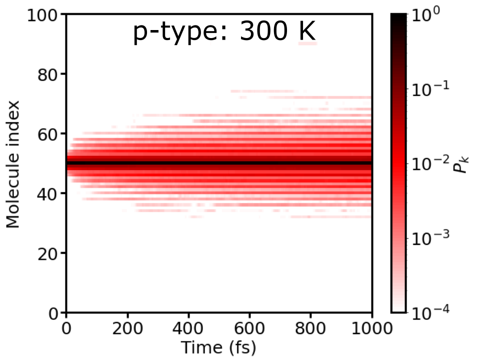

(b)

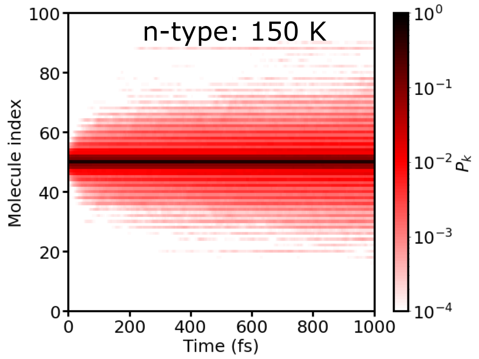

(c)

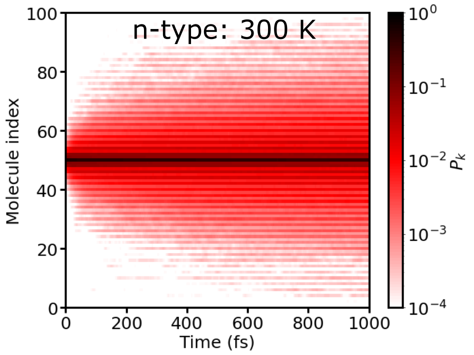

(d)

Supplementary Figure 8: Time-evolved charge density profile as a function of temperature. Time-evolved charge density profile at T = 150 K ((**a**), (**c**)) and 300 K ((**b**), (**d**)) for holes and electrons, respectively. The color scale indicates the log of the normalized charge density averaged over all trajectories. Note how electrons spread more than holes and how the wavefunction spreading is larger at higher temperatures.

References

1. Schott, S. *et al.* Polaron Spin Dynamics in High-Mobility Polymeric Semiconductors. *Nature Physics* **15,** 814 (2019).
2. Su, W. P., Schrieffer, J. R. & Heeger, A. J. Solitons in Polyacetylene. *Physical Review Letters* **42,** 1698–1701 (1979).
3. Dilmurat, R., Prodhan, S., Wang, L. & Beljonne, D. Thermally Activated Intra-Chain Charge Transport in High Charge-Carrier Mobility Copolymers. *The Journal of Chemical Physics* **156,** 084115 (2022).
4. Prodhan, S., Giannini, S., Wang, L. & Beljonne, D. Long-Range Interactions Boost Singlet Exciton Diffusion in Nanofibers of π-Extended Polymer Chains. *The Journal of Physical Chemistry Letters* **12,** 8188–8193 (2021).
5. Brédas, J.-L., Beljonne, D., Coropceanu, V. & Cornil, J. Charge-Transfer and Energy-Transfer Processes in Pi-Conjugated Oligomers and Polymers: A Molecular Picture. *Chemical Reviews* **104,** 4971–5004 (2004).
6. Troisi, A. Dynamic Disorder in Molecular Semiconductors: Charge Transport in Two Dimensions. *The Journal of Chemical Physics* **134,** 034702 (2011).
7. Giannini, S., Ziogos, O. G., Carof, A., Ellis, M. & Blumberger, J. Flickering Polarons Extending over Ten Nanometres Mediate Charge Transport in High-Mobility Organic Crystals. *Advanced Theory and Simulations* (2020).
8. Shuai, Z. Thermal Vibration Correlation Function Formalism for Molecular Excited State Decay Rates. *Chinese Journal of Chemistry* **38,** 1223–1232 (2020).
9. Reimers, J. R. A Practical Method for the Use of Curvilinear Coordinates in Calculations of Normal-Mode-Projected Displacements and Duschinsky Rotation Matrices for Large Molecules. *The Journal of Chemical Physics* **115,** 9103–9109 (2001).
10. Troisi, A. Prediction of the Absolute Charge Mobility of Molecular Semiconductors: The Case of Rubrene. *Advanced Materials* **19,** 2000–2004 (2007).
11. Coropceanu, V. *et al.* Charge Transport in Organic Semiconductors. *Chemical Reviews* **107,** 926–952 (2007).
12. Giannini, S. & Blumberger, J. Charge Transport in Organic Semiconductors: The Perspective from Nonadiabatic Molecular Dynamics. *Accounts of Chemical Research* **55,** 819–830 (2022).
